# Supplementary material for: Changes in data management contribute to temporal variation in gestational duration distribution in the Swedish Medical Birth Registry
Source: PLoS One. 2020 Nov 6;15(11):e0241911. doi: 10.1371/journal.pone.0241911 (PMC7647076; doi:10.1371/journal.pone.0241911)
Supplement: S1 Table — The table shows the year in which there was an observed change in the expected gestational duration. The first column shows the year in which there was a drop from the initial expected duration of 281 to 280 days. The second column shows the year in which there was a drop in the expected duration of 280 to 279 days. Sample size: n = 3 940 577. Analyses were limited to available counties. The counties where a change in the expected gestational duration was not observed during 1983–2012 (such as Uppsala and Kronoberg) are not included. (DOCX) [file pone.0241911.s001.docx]

| Swedish county | drop from 281 to 280 days | drop from 280 to 279 days |
| --- | --- | --- |
| Stockholm | 1984 | - |
| Jönköping | 1985 | 1998 |
| Kronoberg | 1985 | - |
| Östergötland | 1986 | 1991 |
| Halland | 1988 | - |
| Skåne | 1988 | - |
| Norrbotten | 1988 | - |
| Blekinge | 1999 | - |
| Gotland | 1990 | 1991 |
| Dalarna | - | 1989 |
| Jämtland | - | 1989 |
| Värmland | - | 1989 |
| Gävleborg | - | 1990 |
| Västmanland | - | 1998 |
| Västerbotten | - | 1998 |
| Västra Götaland | - | 2005 |
| Västernorrland | - | 2005 |
| Södermanland | - | 2010 |

**S1 Table. Variations in the expected gestational duration among Swedish counties, Swedish Medical Birth Register, 1983**-**2012.**
