# Supplementary material for: Changes in data management contribute to temporal variation in gestational duration distribution in the Swedish Medical Birth Registry
Source: PLoS One. 2020 Nov 6;15(11):e0241911. doi: 10.1371/journal.pone.0241911 (PMC7647076; doi:10.1371/journal.pone.0241911)
Supplement: Attachment — Submitted filename: Response to Reviewers.docx [file pone.0241911.s002.docx]

To the Editorial Team,

PLOS ONE

Gothenburg, October, 2020

Dear Editor Dr. Frank T. Spradley,

Thank you for considering our paper “Changes in data management contribute to temporal variation in gestational duration distribution in the Swedish Medical Birth Registry” for publication in PLOS ONE. We want to thank the reviewer for their comments on our manuscript. The detailed response to all the comments follows below.

Yours sincerely,

on behalf of all authors,

Dominika Modzelewska

Sahlgrenska Academy, University of Gothenburg

Institute of Clinical Sciences

Dept of Obstetrics and Gynecology

SE-405 30 Gothenburg, Sweden

e-mail: dominika.modzelewska@gu.se

*Journal requirements:*

*When submitting your revision, we need you to address these additional requirements.*

*1. Please ensure that your manuscript meets PLOS ONE's style requirements, including those for file naming.*

**Response**: We are grateful for the reviewer’s valuable comments and thank the Editor for the chance to improve our paper accordingly. The following changes were introduced to the manuscript: 1) all the headings of the major sections were adjusted, 2) all the headings of the sub-sections were adjusted accordingly, 3) whole manuscript was double-spaced, 4) references were cited before the punctuation sign, 5) short title was removed from the title page, 6) titles were removed from the author list.

*2. We note that you have indicated that data from this study are available upon request. PLOS only allows data to be available upon request if there are legal or ethical restrictions on sharing data publicly. For information on unacceptable data access restrictions, please see http://journals.plos.org/plosone/s/data-availability#loc-unacceptable-data-access-restrictions.*

*In your revised cover letter, please address the following prompts:*

*a) If there are ethical or legal restrictions on sharing a de-identified data set, please explain them in detail (e.g., data contain potentially identifying or sensitive patient information) and who has imposed them (e.g., an ethics committee). Please also provide contact information for a data access committee, ethics committee, or other institutional body to which data requests may be sent.*

**Response:** The following explanation was introduced to the cover letter: “The Swedish Medical Birth Registry is a national dataset; therefore, it is considered as public property. However, access to the data is given only to the researches with permission from a Swedish regional ethical review board and after approval of the research plan by the data manager. The data request may be sent to The Swedish National Board of Health and Welfare (https://www.socialstyrelsen.se/)”.

*b) If there are no restrictions, please upload the minimal anonymized data set necessary to replicate your study findings as either Supporting Information files or to a stable, public repository and provide us with the relevant URLs, DOIs, or accession numbers. Please see http://www.bmj.com/content/340/bmj.c181.long for guidelines on how to de-identify and prepare clinical data for publication. For a list of acceptable repositories, please see http://journals.plos.org/plosone/s/data-availability#loc-recommended-repositories.*

*We will update your Data Availability statement on your behalf to reflect the information you provide.*

*Reviewers' comments:*

*Reviewer's Responses to Questions

1. Is the manuscript technically sound, and do the data support the conclusions?

The manuscript must describe a technically sound piece of scientific research with data that supports the conclusions. Experiments must have been conducted rigorously, with appropriate controls, replication, and sample sizes. The conclusions must be drawn appropriately based on the data presented.*

*Reviewer #1: Yes*

*2. Has the statistical analysis been performed appropriately and rigorously?*

*Reviewer #1: Yes*

*3. Have the authors made all data underlying the findings in their manuscript fully available?
Reviewer #1: Yes*

*4. Is the manuscript presented in an intelligible fashion and written in standard English?
Reviewer #1: Yes*

*5. Review Comments to the Author*

***Reviewer #1 comment #1:*** *Very important study showing that population variability of gestational duration is largely affected by changes in obstetrical practice, estimation methods, and registration methods. It shows eloquently the importance of increasing granularity of gestational age to days, which in Sweden is available since last century, using a combination (LMP, US) of data of the birth registry. This could be a great inspiration for other countries. Only when you overcome the binary term/preterm you can see more granular.*

**Response:** We thank the reviewer for her very supportive feedback. We also agree with and support the “granular approach” towards gestational duration. Working with binary (preterm, term) loses information and hides the stories behind the data. This is particularly the case in obstetrics where the risk curve is often parabolic in nature.

***Reviewer’s comment #2:*** *It also provides clever alternatives for visualizing data, the last graphic with the variation of GS in days, including leap years is one them.*

**Response:** We thank the reviewer for her feedback. Communication is very important for us, and we find graphics convey a lot of information easily. We work towards better graphical data exploration and visualization. Every comment and suggestion on how to improve are very welcome.

***Reviewer’s comment #3:*** *The idea of having a reference for "expected gestational duration" and monitoring its trends in term period sounds very obvious but very under-explored.*

**Response:** We thank the reviewer for sharing that observation. We also agree that sometimes very obvious things are left unexplored. That is why our work (research) never finishes.

***Reviewer’s comment #4:*** *Some small suggestions: When referring to "gestation duration mode" it would be best described by gestational duration estimates local cultures, as the author further explains?*

**Response:** We understand that the reviewer is referring to Figure 3, of which, the caption includes the phrase “gestational duration mode”. The word “mode” refers to the statistical measure, the value that occurs the most frequently in the dataset. However, in gestational duration distribution we observed multiple gestational durations that occur more frequently than the neighbouring values. Such observation we called “local modes”. The phrase “gestational duration mode” refers to that distributional characteristic.

In order to reduce the ambiguity and felicitate the understanding, we rephrased “gestational duration mode” and “local modes” to “gestational duration distribution” and “peaks”, respectively. Figure’s caption and its detailed explanation were changed: “Fig 3. Gestational duration distribution, Swedish Medical Birth Register, 1973–2012. A) Gestational duration distribution. Gestational durations that occurred at higher frequency than expected (peaks) are marked by solid black lines. Sample was reduced to pregnancies registered from 1973 to 1982, that is, to the period in which peaks were most common. Adjacent gestational durations to peaks are marked by black dashed lines. B) Average ratio of peaks’ frequency to the mean of adjacent gestational duration frequency from 1973 to 2012. Sample size: n = 3 940 577.”, lines 192-198, page 9.

***Reviewer’s comment #5:*** *The effect of obstetric interventions could be a little more explored.*

**Response:** We thank the reviewer for inspiring further exploration of the topic. However, the aim of this paper was slightly different. In this work, we wanted to show that there are many different types of variables affecting the observations of gestational duration. The multifactorial nature of gestational duration is an obvious and often stated fact. However, we have noticed that there is a tendency in restricting the focus to biological factors. In this paper, we wanted to show that other, technical factors contribute to the observed gestational duration variability as well. Therefore, we did not intend to explore and detect all the factors contributing to the variability. We wanted to present and remind about the variability in the subgroups of the factors that affect the observations and contribute to the gestational duration variability.

Our research group explores genetic and environmental contributions to gestational duration. In our work, we see that it is very crucial to keep in mind that observed variability in gestational duration is a result of different subgroups of factors. Considering only one group of factors may lead to possible problems of interpretation. Estimates of the association studies might contain the effects of all variables. Emphasizing the complexity of the problem will inspire even more scrupulous study designs and careful interpretation of the obtained estimates.

***Reviewer’s comment #6:*** *A hypothesis: are ultrasound measures so uniform because there was a recalibration of growth/gestational age curves during the period?*

**Response:** Over the years, gestational duration estimation approach based on the ultrasound examination has been adjusted once and that was from a report published in 2010 (1). Before 2010, the most common reference used was from Person et al 1986 (2). In 2010, Swedish Association for Obstetricians and Gynaecologists released the guidelines for pregnancy dating. In general, the adjustments relate to the timing of the ultrasound examination (first, second trimester), biometric parameters (crown–rump length, biparietal diameter), fetal sex.

***Reviewer’s comment #7:*** *Finally, reading it from São Paulo, Brazil, where the mean GA is 273, and for cesareans in the private sector is 268, it is great to see that in some regions in Sweden it is still 282, but at least 278. Adding granularity to GA is so feasible, so explanatory that should be widely available data. This is why I just approve the paper and I am eager to see it published.*

**Response:** We thank the reviewer for your time and the approval.

*Availability of data: under conditions.
English: very good for my non-native eyes.*

*6. PLOS authors have the option to publish the peer review history of their article. If published, this will include your full peer review and any attached files. If you choose “no”, your identity will remain anonymous but your review may still be made public. Do you want your identity to be public for this peer review?*

*Reviewer #1: Yes: Carmen Simone Grilo Diniz*

**References:**

1.	Kullinger M, Granfors M, Kieler H, Skalkidou A. Adherence to Swedish national pregnancy dating guidelines and management of discrepancies between pregnancy dating methods: a survey study. Reprod Health

2.	Persson PH, Weldner BM. Reliability of ultrasound fetometry in estimating gestational age in the second trimester. Acta Obstet Gynecol Scand. 1986;65(5):481–3.
